# Theta-burst rTMS in schizophrenia to ameliorate negative and cognitive symptoms: a double-blind, sham-controlled, randomized clinical trial

**DOI:** 10.1192/j.eurpsy.2024.455

**Published:** 2024-08-27

**Authors:** B. Orban Szigeti, K. Farkas, L. Herman, R. Zsigmond, J. Rethelyi, G. Csukly

**Affiliations:** ^1^Psychiatry, Semmelweis University, Budapest, Hungary

## Abstract

**Introduction:**

Schizophrenia is a major mental disorder that affects approximately 1% of the population worldwide. Social cognition impairments and negative symptoms such as blunted affect or emotional withdrawal strongly contribute to the psychosocial functioning deficits and long-term disability in schizophrenia. The state-like and trait-like components of social cognition are impaired in schizophrenia

**Objectives:**

Treatment effects of conventional approaches with antipsychotics or psychosocial interventions are limited when it comes to reducing negative and cognitive symptoms in schizophrenia. While there is emerging clinical evidence that new, augmented protocols based on theta-burst stimulation can increase rTMS efficacy dramatically in depression, data on similar augmented therapies are very limited in schizophrenia. The different patterns of network impairments in subjects may underlie that some but not all patients responded to given stimulation locations.

**Methods:**

Therefore, we propose an augmented theta-burst stimulation protocol in schizophrenia by stimulating both locations connected to negative symptoms, namely the vermis of the cerebellum and the left Dorsolateral Prefrontal Cortex (DLPFC). Ninety subjects with schizophrenia presenting negative symptoms and aging between 18-50 years will be randomized to active and sham stimulation in a 1:1 ratio. The TBS parameters we adopted follow the standard TBS protocols, with 3-pulse 50-Hz bursts given every 200 ms (at 5 Hz) and an intensity of 100% active motor threshold. We plan to deliver 1800 stimuli to the vermis and 1800 stimuli to the left DLPFC daily in two 9.5-minute blocks for four weeks.

**Results:**

The primary endpoint is the change in negative symptom severity measured by the Positive and Negative Syndrome Scale (PANSS). Secondary efficacy endpoints are the change in cognitive flexibility measured by the Wisconsin Card Sorting Test and the change in social cognition assessed by the ‘Reading the Mind in the Eyes’, facial emotion recognition, and the ‘Faux pas’ tests. The safety outcome is the number serious adverse events.

**Conclusions:**

In conlucion the aim of our study is to proove the safety and efficacy of theta burst stimulation for treating negativ sympotoms of schizophrenia.

**Disclosure of Interest:**

None Declared

